# Clinical and laboratory evaluation of new immigrant and refugee children arriving in Greece

**DOI:** 10.1186/s12887-017-0888-7

**Published:** 2017-05-26

**Authors:** Ioanna D. Pavlopoulou, Marsela Tanaka, Stavroula Dikalioti, Evangelia Samoli, Pavlos Nisianakis, Olga D. Boleti, Konstantinos Tsoumakas

**Affiliations:** 1 Faculty of Nursing, Paediatric Clinic, P. & A. Kyriakou” Children’s Hospital, National and Kapodistrian University of Athens, 123 Papadiamantopoulou str, 11527 Athens, Greece; 2 Faculty of Nursing, Postgraduate Program, National and Kapodistrian University of Athens, 123 Papadiamantopoulou str, 11527 Athens, Greece; 3 Department of Hygiene, Epidemiology and Medical Statistics, National and Kapodistrian University of Athens, Medical School, 75 M. Asias str, 11527 Athens, Greece; 4 Center of Biological Research of Armed Forces, 414 Military Hospital, I. Velliou str, 15236 Athens, Greece

**Keywords:** Health status, Migrant children, Refugees, Vaccination, Tuberculosis, Hepatitis B virus, Blood lead levels

## Abstract

**Background:**

Migrant children are a population at risk for various health problems. Despite the increased inflow of migrants in Greece, data regarding their health assessment are lacking. This study aims to describe the clinical and certain laboratory characteristics and identify possible associations in a group of new immigrant (I) and refugee (R) children, arriving in Athens, Greece.

**Methods:**

A prospective, cross- sectional study was performed in a migrant outpatient clinic of a tertiary Children’s hospital. All immigrant and refugee children, examined to obtain a health certificate, within 3 months of their arrival in the country, were enrolled. Clinical and laboratory information was collected in a pre- designed form. We applied multiple logistic regression models to investigate the association between the child’s status (immigrant vs refugee) and health indicators controlling for possible confounding effects, mainly of age and area of origin.

**Results:**

From 2010 to 2013, a total of 300 children (I/R:138/162) with a mean age of 7.08 (range 1–14) years were included. Overall, 79.3% presented unknown vaccination status, 21.3% dental and 7.3% additional clinical problems. Latent tuberculosis was identified in 2.7%, while anemia, low serum ferritin and eosinophilia were found in 13.7%, 17.3%, and 22.7% of subjects, respectively. 57.7% had protective antibodies to hepatitis B surface antigen (anti-HBs ≥ 10 IU/L) and 30.6% elevated blood lead levels (EBLLs). Immigrants had less likely unknown immunization (OR = 0.25, *p* < 0.001), but had increased odds of low ferritin (OR = 1.97, *p* = 0.043), EBLLs (OR = 2.97, *p* = 0.001) and protective anti-HBs (OR = 1.79, *p* = 0.03). Age was inversely associated with anemia (OR = 0.0.89, *p* = 0.017), low ferritin (OR = 0.91, *p* = 0.027), EBLLs (OR = 0.86, *p* = 0.001) or positive anti-HBs (OR = 0.92, *p* = 0.025). Children from Europe or Africa presented decreased probability of EBLLs (OR = 0.31, *p* = 0.001, and OR = 0.15, *p* = 0.005, respectively) compared to those from Asia.

**Conclusions:**

New immigrant and refugee children presented distinct clinical problems and certain laboratory abnormalities. Some of these health issues differed according to their migration status, age and geographic area of origin. These findings provide evidence that may assist the optimal approach of this vulnerable population.

## Background

Migrant children represent a population at risk for a variety of physical and mental health problems as a result of their limited access to quality health care, the increased prevalence of infectious diseases in their countries of origin and the suboptimal conditions during the process of migration [1]. These include malnutrition and secondary nutritional deficiency diseases, lead poisoning, various infections and transmissible diseases as well as psychiatric disorders, the latter as a result of stress [2–8].

Among the population of migrant children, refugees represent a group of higher risk for the aforementioned health issues because of the nature of life-threatening experiences before and during flight from their home countries as well as the difficult circumstances of existence in exile [7, 9]. Although migrant children in general do not pose an imminent health threat to their host countries upon arrival, it is evident that a health assessment is important since the majority of the above conditions are treatable, and if undiagnosed, may result in serious adverse health consequences. Therefore, screening programs at entry are in place in many countries around the world [1, 10].

The proportion of migrants in European population is substantial and continues to grow despite an initial slowdown following the global economic crisis [11]. Moreover, Europe has been facing lately an increased inflow of refugees entering through Southern Mediterranean countries, partly as a result of the changing dynamics in the Middle East [12, 13]. However, information about the health of migrants in Europe is limited and inconclusive, due to the heterogeneity and small size of this population, more so data regarding children [11]. As a result, the optimal way to screen new migrants and what to screen for, remains an ongoing debate among European countries and approaches vary considerably [14–16].

For the past two decades Greece has experienced an increased inflow of migrants, mainly economic immigrants from Eastern European countries. According to 2011 Census data, a total of 912.000 immigrants with a residence permit were documented, comprising approximately 9% of the country’s population. Of these, 203.693 were children and adolescents between 0 and 19 years of age [17]. Furthermore, between the years 2010 and 2013, statistic data on illegal immigration have documented 253.104 apprehensions of irregular migrants at the borders and within the country with an estimated 30% being children, these numbers increasing ever since [18]. Until now, no special screening strategy has been implemented, and children of immigrant parents receive a clinical examination, chest radiography and tuberculin testing in order to receive a green card. The same approach applies for children of asylum seekers before their placement in shelters [19].

In response to the paucity of information in this area, we sought to describe the demographic, clinical and certain laboratory characteristics and to identify possible determinants among newly arriving immigrant and refugee children in our country.

## Methods

### Study population

All immigrant and refugee children, who received a health status evaluation at a special outpatient clinic, between May 2010 and March 2013, within 3 months of their arrival in the country, were eligible for participation in this cross-sectional study. This migrant clinic is located at “P. & A. Kyriakou” Children’s Hospital, one of two largest tertiary pediatric hospitals in Athens, Greece. It started its operation in 2010, in response to the increasing migratory flow in our country, aiming to identify the major health needs of this population and provide evidence for its optimal approach and management. Immigrant children are self- referred to this clinic for a health evaluation in order to obtain a green card, while refugees are referred by collaborating non-governmental organizations and social services, before their resettlement in shelters.

Demographic data including date of birth, gender, and country of origin, as well as additional information concerning past medical and family history and date of entry in this country, were obtained from parents or guardians, from travel and medical documents, while vaccination history from immunization cards, when available. In children with missing immunization records, the presence of the characteristic scar over the deltoid area was accepted as evidence of BCG vaccination. All children received a complete physical examination, including anthropometric measurements and calculation of their body mass index (BMI). Consent was obtained by all parents or guardians before laboratory investigation. Those parents who did not speak Greek or English were informed regarding the aim of the study through interpreters, when present, or through waivers issued in their native language. Further follow up appointments were scheduled to address any clinical or laboratory issues that would arise. The study protocol was approved by the institution review board of “P. & A. Kyriakou” Children’s Hospital.

### Glossary

For the purpose of this study, “immigrants” were defined as the children of parents with long- term residence permit, entering this country for family reunification, while as the remaining, including refugees, asylum seekers or irregular migrants were defined as “refugees”. Together, immigrants and refugees were defined as “migrants”. The above terms are in agreement with those used by the International Organization for Migration (IOM) in the Glossary of Migration [20].

### Laboratory evaluation

All participants underwent tuberculosis screening, including a Mantoux test (purified protein derivative) and a chest radiograph (CXR). Furthermore, blood samples were obtained, and the following laboratory evaluation was performed: Full blood count, serum ferritin levels and serologic markers against hepatitis B (HBV) and hepatitis C (HCV) virus. More specifically, the levels of immunoglobulin G (IgG) antibodies against hepatitis B surface antigen (anti-HBs) were measured by use of AxSYM AUSAB Reagent Kit, Calibrators, and Controls (Abbott Laboratories). In addition, serum samples were tested for antibodies against hepatitis B core antigen, by use of AxSYM CORE (Abbott Laboratories), and for hepatitis B surface antigen titers, by use of AxSYM HBsAg (V2) (Abbott Laboratories), to distinguish between undocumented immunization and a state of infection or carriage. Furthermore, whole blood (EDTA) samples were stored at 4–6 °C for measurement of blood lead levels (BLL) by inductively coupled plasma- mass spectrometry ICP-MS (Agilent 7700×–Agilent Technologies, Waldbronn, Germany) at a later stage.

### Interpretation of laboratory results

Anemia was defined as hemoglobin levels of less than 11 g/dl, less than 11.5 g/dl, and less than12 g/dl at the age groups of 12–59 months, 5–11 years and 12–14 years, respectively, low serum ferritin as levels of less than 12 ng/ml, eosinophilia as eosinophil count of >450/mm^3^and elevated blood Lead if respective levels were higher than 5 μg/dL [21]. Serologic immunity to hepatitis B virus was assumed if serum hepatitis B surface antigen antibody levels were 10 IU/L or higher. Tuberculin testing was considered positive at 10 mm or more of induration, irrespective of previous vaccination with BCG, in the absence of other high-risk criteria [22].

### Statistical analysis

The statistical analyses were conducted using the SPSS statistical package (IBM Statistical Package for Social Sciences v. 19.0, Chicago, Illinois, USA). At first, we distributed children by immigrant or refugee status according to their demographic characteristics and medical history as well as the levels of the studied compounds. The statistical significance of the observed differences by status was estimated by use of the t-test for continuous variables or the X^2^ test (or Fisher’s test) for categorical variables. Consequently, we investigated the association between high versus low lead levels according to migration status, age, and country of origin. *P* < 0.05 was considered to indicate statistical significance.

Finally, we applied multiple logistic regression models to investigate the association between the child’s migration status (immigrant versus refugee) with the main health indicators: anemia (yes versus no), elevated BLL (yes versus no), tuberculin test equal or higher than 10 mm (yes versus no), immunization status (unknown versus known) and anti-HBs (positive versus negative). In all models, we controlled for the child’s age (continuously, in years) and geographic area of origin (as categorical variable with 3 levels, where 0 = Asia, 1 = Europe, 2 = Africa), except for the association with tuberculin testing for which we only controlled for the child’s age, due to the extremely small number of cases, the vast majority of which originated from Africa (86%). For the association with anti-HBs, we additionally controlled for the child’s BMI continuously, in kg/m^2^.

## Results

### Demographics

**Fig. 1:**
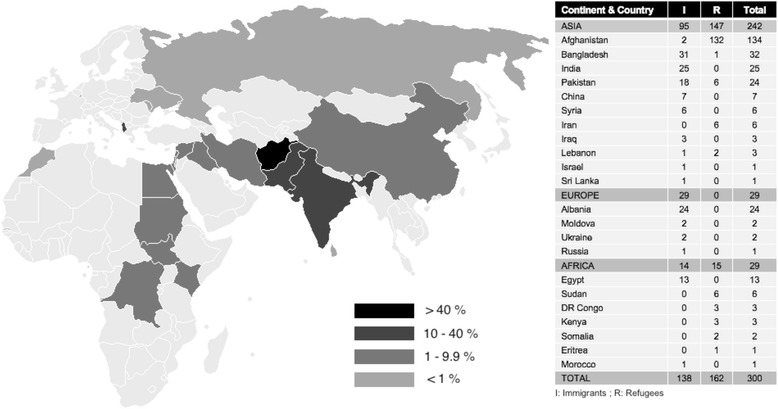
Distribution of new migrant children according to continent, country of origin and migration status. Adapted from original uploader: Roke (https://commons.wikimedia.org/wiki/File:BlankMap-World-v2.png), colour by present percentage of migrants according to country, Creative Commons Legal Code

Between May 2010, and March 2013, a total of 300 newly arrived immigrant (*N* = 138, 46%) and refugee children (*N* = 162, 54%), were recruited (mean age 7.1 years old, range 1–14 years). As shown, the majority originated from Asia (80.7%), and the most common countries of birth were Afghanistan (44.6%) and Bangladesh (10.7%). Most immigrant children originated from Bangladesh, whereas refugee children from Afghanistan (Fig. 1).

### Vaccination status

As illustrated in Table 1, the great proportion of migrant children overall, presented unknown vaccination status (79.3%) and this was more prominent among the group of refugees (*R* = 91.3% versus *I* = 65.2%, *p*-value < 0.001). BCG vaccination, identified through scarring and/or vaccination records, was evidenced by the majority (87.3%) of children, more so among refugees (*p*-value = 0.055).

**Table 1:** Characteristics of newly arriving immigrant and refugee children, (*n* = 300)

	Total *N* (%)	I *N* = 138 (46)	R *N* = 162 (54)	*p-value*
*Gender*				
M/F	176/124 (58.7/41.3)	86/52 (62.3/37.5)	90/72 (55.6/44.4)	0.236
*Mean age* (SD), years	7.08 (3.8)	6.3 (3.8)	7.8 (3.7)	0.001
*Geographic area of origin*				
Europe	29 (9.7)	29(21.0)	0 (0.0)	<0.001
Africa	29 (9.7)	14 (10.1)	15 (9.3)	
Asia	242 (80.7)	95 (68.8)	147 (90.7)	
*BMI* (Median (IQR)^a^		15.7 (2.7)	15.5 (3.1)	0.886
*Anemia* ^b^	41 (13.7)	21 (15.2)	20 (12.3)	0.470
*Pb* (≥5 μg/ dl)^c^	72 (30.6)	45 (37.2)	27 (23.7)	0.025
*Ferritin* (≤12 ng/l)	50 (17.3)	30 (22.1)	20 (13.1)	0.044
*Eosinophilia* (≥450mm^3^)	68 (22.7)	35 (25.4)	33 (20.4)	0.303
*Vaccination status* (unknown)	238 (79.3)	90 (65.2)	148 (91.4)	<0.001
*BCG* (records/scar)	262 (87.3)	115 (83.3)	147(90.7)	0.055
*Mantoux* ≥ 10 mm	8 (2.7)	0(0.0)	8 (4.9)	0.008
*HBV serology*				
anti-HBs (+)	173 (57.7)	90 (65.2)	83 (51.2)	0.015
anti-c (+)	0 (0.0)	0 (0.0)	0 (0,0)	
HbsAg (+)	0 (0.0)	0 (0.0)	0 (0,0)	
*HCV serology*				
anti- HCV (+)	1 (0.6)	0 (0.0)	1 (0.6)	0.365

*p*- values from t-test for continuous variables and X^2^ or Fisher’s exact test for categorical variables

*anti-HBs* IgG antibodies against hepatitis B surface antigen, *anti-HBc* IgG antibodies against hepatitis B core antigen, *HBsAg* hepatitis B surface antigen, *anti- HCV* IgG antibodies against hepatitis C virus

^a^ *BMI* Body mass index, *IQR* Interquartile range

^b^Definition of anemia by age group: Hb <11 mg/dl (12–59 months); Hb <11.5 mg/dl (5–11 years); Hb <12 mg/dl (12–14 years)

^c^Pb: Blood lead levels; a total of 235 children were tested

### Clinical findings

Following clinical examination, dental abnormalities, especially carries, was the most frequent clinical problem identified (21.3%; *I* = 17.4% versus *R* = 24.7%, *p*-value = 0.124), while as other clinical conditions requiring intervention were present in 7.3% of the total study population. These included respiratory and skin infection (*n* = 2), genitourinary (*n* = 5) or cardiological (*n* = 6) problems, thyroid disease (*n* = 2), hearing (*n* = 1), skeletal abnormalities (*n* = 2), bone fracture (*n* = 1) and neurological/hearing problems (*n* = 3).

### Laboratory screening

As demonstrated in Table 1, anemia was present in 13.7% (*I* = 15.2% versus *R* = 12.3%, *p*-value = 0.470) and low serum ferritin in 17.3% of subjects (*I* = 22.1% versus *R* = 13.1%, *p*-value = 0.044). Eosinophilia was found in 22.7% (*I* = 25.4% versus *R* = 20.4%, *p*-value = 0.303) of migrant children. Nearly one-third of the whole study population had BLLs ≥5 μg/dL, and this was more prominent among the immigrant group (*I* = 37.2% versus *R* = 23.7%, *p*-value = 0.025). Blood Lead levels ranged from 0.7 to 21.03 μg/dL (mean 4.3 μg/dL, median 3.55 μg/dL) in both immigrant and refugee children, and the highest value (21.3 μg/dL) was detected in a 3-year old immigrant boy from Pakistan. The characteristics of children according to low or elevated BLLs are presented in Table 2. As shown, almost all individuals with EBLLs originated from Asia (*n* = 68, 94.4%), mainly Afghanistan (*n* = 27, 37.5%), Bangladesh (*n* = 18, 25%), Pakistan (*n* = 12, 16.7%) and India (*n* = 8, 11.1%), (*p*-value < 0.001). It is noteworthy that, more than half of the children with EBLLs belonged to the 1–5 year age group. Anemia was not associated with EBLLs, as opposed to iron depletion, expressed as low ferritin levels, where the above association was statistically significant (*p*-value = 0.023).

**Table 2:** Blood lead levels according to population characteristics

	Blood Lead levels^a^ μg/dl		*p-value*
	<5 *n* = 163 (69.4%)	≥5 *n* = 72 (30.6%)	
*Migration status*			0.025
Immigrants	76 (46.6)	45 (62.5)	
Refugees	87 (53.4)	27 (37.5)	
*Age group* (years)			0.001
1–5	58 (35.6)	42 (58.3)	
6–14	105 (64.4)	30 (41.7)	
*Geographic area of origin*			<0.001
Europe	27 (16.6)	1 (1.4)	
Africa	20 (12.3)	3 (4.2)	
Asia	116 (71.2)	68 (94.4)	
*Anemia* ^b^	23 (14.1)	10 (13.9)	0.964
*Ferritin* (≤12 ng/l)	21 (13.2)	18 (25.4)	0.023

^a^n = a total of 235 children were tested

^b^Definition of anemia by age group: Hb <11 mg/dl (12–59 months); Hb <11.5 mg/dl (5–11 years); Hb <12 mg/dl (12–14 years)

### Infectious diseases

Eight out of 300 children (2.7%), all refugees, had a positive Mantoux test. Two of them originated from Congo and the remaining six from Afghanistan. No abnormality was detected on their CXR and all were vaccinated with BCG. We were only able to perform a QuantiFERON test (confirming infection) in the two patients from Congo.

No child was positive against hepatitis B surface antigen, and protective antibodies to HBV surface antigen (anti-HBs ≥ 10 IU/L) were detected in 173 (57.7%) [*I* = 65.2% versus *R* = 51.2%, p- value 0.015] of all children. These were considered to be immunization acquired since no child tested positive for antibodies against hepatitis B core antigen (Table 1).

In Table 3 the odds ratios (OR) and corresponding 95% confidence intervals (CIs) for the associations between anemia, low ferritin levels, elevated BLLs, positive Mantoux test, unknown immunization status and serologic immunity against hepatitis B, in terms of positive anti-Hbs, with the child’s migration status, age and geographic area of origin are presented. As shown, immigrants had significantly increased odds of lower ferritin levels, EBLLs and positive anti-HBs. Specifically, immigrant status presented a statistically significant association with low ferritin levels (OR = 1.97 *p*-value = 0.043), EBLLs (OR = 2.97, *p*-value = 0.001) and positive anti-HBs (OR = 1.79, *p*-value 0.030). Moreover, immigrants were less likely to have unknown immunization status (OR = 0.25, *p* < 0.001) while there was an indication for decreased odds for positive tuberculin testing that did not reach statistical significance possibly due to the small sample size. Age was inversely associated with anemia (OR = 0.89, *p*-value = 0.017), lower ferritin levels (OR = 0.91, *p*-value = 0.027), EBLLs (OR = 0.86, *p*-value = 0.001) or positive anti-HBs (OR = 0.92, *p*-value = 0.025). Children from Europe or Africa presented decreased probability of EBLLs (OR = 0.31, *p* = 0.001, and OR = 0.15, *p* = 0.005, respectively) compared to those from Asia.

**Table 3:** Multiple logistic regression derived odds ratios (and 95% confidence intervals) for the risk of presence of anemia, low serum ferritin, elevated Pb, Mantoux ≥10 mm, unknown immunization status and anti-HBs (+ vs -) and possible determinants

	*Anemia Hb* ^a^		*Ferritin* (≤12 ng/l)		*Elevated Pb* ^c^ (≥5 vs <5 μg/dl)		*Mantoux* ≥ 10 mm	*Immunization status* (Unkown vs known)	*anti-HBs* ^d^ (+ vs -)			
	*OR (95% CI)*	*p-value*	*OR (95% CI)*	*p-value*	*OR (95% CI)*	*p-value*	*OR (95% CI)*	*p-value*	*OR (95% CI)*	*p-value*	*OR (95% CI)*	*p-value*
*Migration status*												
Refugee	Reference category											
Immigrant	1.29 (0.64–2.61)	0.482	1.97 (1.02–3.81)	0.043	2.97 (1.58–5.60)	0.001	0 (0.00- n.e)	0.996	0.25 (0.12–0.51)	<0.001	1.79 (1.06–3.01)	0.030
*Age* (per 1 year)	0.89 (0.81–0.98)	0.017	0.91 (0.83–0.99)	0.027	0.86 (0.79–0.94)	0.001	0.96 (0.79–1.16)	0.653	1.03 (0.95–1.12)	0.506	0.92 (0.86–0.99)	0.025
*Geographic area of origin*												
Asia	Reference category											
Europe	0.35 (0.08–1.63)	0.181	0.36 (0.10–1.32)	0.125	0.31 (0.00–0.24)	0.001	-		0.25 (0.10–0.59)	0.002	0.65 (0.28–1.53)	0.325
Africa	0.62 (0.17–2.20)	0.457	0.62 (0.20–1.95)	0.417	0.15 (0.04–0.56)	0.005	-		2.14 (0.60–7.67)	0.244	2.14 (0.88–5.19)	0.092
*BMI* ^b^(per 5 kg/m^2^)	-	-	-	-	-	-	-	-	-	-	1.56 (1.04–2.33)	0.031

^a^Definition of anemia by age group: Hb <11 mg/dl (12–59 months); Hb <11.5 mg/dl (5–11 years); Hb <12 mg/dl (12–14 years)

^b^ *BMI* body mass index

^c^Pb: Blood Lead levels; *n* = 235 children were tested

^d^anti-HBs: IgG antibodies against hepatitis B surface antigen

## Discussion

The present study provides evidence on the overall health status of newly arrived immigrant and refugee children attending a special outpatient clinic in the municipality of Athens, Greece. According to our results, nearly one-third of this population presented clinical problems requiring intervention while the majority lacked proof of immunization. Furthermore, certain laboratory abnormalities were noted, including lack of serologic protection against hepatitis B virus, elevated blood lead levels, eosinophilia, anemia and low ferritin. Prevalence of latent tuberculosis was low, and no child suffered chronic hepatitis B or C infection. Some of the clinical and laboratory findings were associated with age, geographic area of origin and migration status.

Among the clinical findings, dental problems, was the most frequently reported health issue with dental caries identified in 21% of the children examined, all of which were referred to an adjacent outpatient dental clinic located at the Dental School of Athens. Oral health is a common area of unmet need among migrant children [23, 24]. Additional clinical problems, including skin, respiratory and surgical conditions have been described frequently by previous investigators, especially among newly arriving refugee children [9].

We found that 80% of our study individuals had unknown vaccination status, the latter being more prominent among the group of refugees. In an attempt to control the transmission of vaccine- preventable diseases among migrant children and their secondary spread to the indigenous population, the lack of immunization proof could be addressed through the initiation of age- appropriate vaccination from the start or by the performance of serologic testing to confirm pre-existing immunity. The latter is an expensive and often impractical strategy but could be considered for hepatitis B, especially for children originating from countries with increased prevalence of chronic HBV infection or for those that may have completed the full 3-dose schedule despite the lack of proof [25].

Screening for infectious diseases revealed that, none of our participants suffered past or chronic hepatitis B infection, and eight were considered to have latent TB. The lack of chronic hepatitis B infection is an important finding as it is this group of patients that will cause an economic burden to the healthcare system of the country of their final resettlement. Interestingly, only slightly more than half of the children presented serologic protection against hepatitis B (anti-HBs ≥10 IU/L) due to vaccination while the remaining were anti-HBs negative. We considered that only a certain proportion of the seronegative children would be unvaccinated against HBV as this vaccine is covered in most countries by the Expanded Immunization Programme with support from GAVI. Therefore, the plausible explanation for this finding is either that these children were partially immunized, or that they presented a natural decline of their antibody titer over time following complete vaccination [26, 27]. To address this problem, a booster dose could be administered to document seroconversion indicating immunologic memory, and if not, continue with two additional vaccine doses, or the individual should be considered non-immune and be vaccinated from the start [28].

Regarding tuberculosis screening, 87.3% of the overall population (83.3% I and 90.7% R) had been vaccinated with BCG. Only eight refugee children (2.7%) had a positive Mantoux ≥10 mm reaction, all were previously vaccinated with BCG and all had a normal CXR. Two of them originated from Congo while the remaining six from Afghanistan, both countries with a high prevalence of tuberculosis where respective immunization has been provided under the GAVI program [29]. As known, BCG vaccination may cause a false-positive result following Mantoux testing that can be clarified through interferon-gamma release assays (IGRAs). However, performance of the latter is expensive, often impractical and may not provide reliable results in very young children [10, 30]. The aforementioned children were managed as suffering latent tuberculosis infection according to international recommendations concerning tuberculosis screening of migrant children [10, 30]. Nevertheless, it is worth mentioning that we were able to perform and subsequently confirm latent tuberculosis infection by QuantiFERON-TB test only in two out of our eight cases (2 children from Congo). The incidence of latent TB among our study participants is much lower than that reported in previous studies concerning migrant children performed in the United States, New Zealand or Australia with the lowest rate (15%) described among refugee children under 5 years of age arriving in New Zealand [9, 31–33]. It is evident that comparison of the above data may be misleading considering that the countries of origin of the migrants are dissimilar to ours and that these studies were conducted at earlier periods.

Additionally, overall 13.7% of our study population (15.2% of immigrant and 12.3% of refugee children) presented with anemia. Rates of anemia have ranged from 12% to 55% according to previous investigators [8, 33–35] depending on the migrants’ native country. Herein low ferritin levels were observed in 17.3% of the overall population and were more frequent among the group of immigrants (22.1% vs. 13.1%; *p* = 0.044). Age was inversely related to the presence of anemia and iron deficiency, supporting the notion that decreased iron intake during the critical period of human growth and development may have contributed to this finding. Immigrants and refugees originating from regions with limited access to iron- rich foods and higher rates of infectious diseases are at risk for iron deficiency [10] and resulting anemia [34]. Despite the fact that low ferritin levels are commonly used as an indicator of iron deficiency, this alone may underestimate the problem since occasional underlying infections falsely increase ferritin values to normal [9].

Eosinophilia is a common finding among immigrants and refugees from parasite-endemic regions [36] and was noted in 22.7% of our study population. In migrant children, infection with helminthic parasites is the commonest cause of eosinophilia [37]. However, aetiologic diagnosis of gastrointestinal parasitosis may prove difficult as many individuals are asymptomatic, and stool parasitology may be negative during the pre-patent phase of the infection. At the same time, even in subjects presenting in the post-patent period, a negative stool investigation does not always preclude infection, since methods of parasite detection are often insensitive, and recognition of ova, cysts or adult parasites in stool largely depends on the experience of the examining scientist and the laboratory technique employed [38]. Furthermore, investigation of parasitic infection requires direct examination of multiple stool samples, collected several days apart, in addition to a stool culture for *Strongyloides*, serology for both *Strongyloide*s and *Schistosoma*, urine microscopy for *Schistosoma* and filarial serology depending on the migrant’s country of origin [37, 38]. Collectively considering the above obstacles, some experts recommend presumptive treatment with albendazole of all asymptomatic migrant children presenting with eosinophilia, who originate from endemic countries, as it is a more feasible and cost-effective approach [37]. For practical and logistic reasons we were only able to obtain a total of twenty single stool samples for microscopy from sixty-eight participants with eosinophilia at the time of the present study, and all yielded negative results. It is evident that this precludes any conclusions regarding the prevalence of parasitic infections in our population. It is worth mentioning though that, stool specimens obtained according to recommendations that were subsequently tested by microscopy, revealed positive findings in five out of one-hundred new migrant children attending our clinic during 2014–2015 regardless of eosinophilia (personal data on file).

Moreover, we identified EBLLs in 30.6% of all children, an alarming rate considering the neurotoxic potential of Lead, its negative impact on cognitive function and attention span [21, 39–41]. Due to accumulating evidence regarding the significant and irreversible adverse effects of even low levels of circulating blood Lead, the Centers for Disease Control and Prevention (CDC) decreased in 2012 the respective “reference level” to 5 μg/dL, stating however that no level of Lead is considered safe. In addition, the importance of primary prevention was highlighted, a revision also endorsed by the American Academy of Pediatrics [21]. Considering that culture-specific lead exposures such as through eye cosmetics or through flaking of lead-based paint may persist even in the host country, the CDC recommends that BLLs should be evaluated in all refugee children within 90 days of their arrival and repeated within 3–6 months after resettlement, regardless of the initial laboratory findings [7]. Generally, EBLLs, among other factors, have been linked to low socioeconomic status, living in buildings with flaking lead-based paint, environmental exposure to lead through industry waste and leaded gasoline but also with malnourishment and micronutrient deficiency, including low iron levels [21, 42, 43]. Indeed in our study, EBLLs were associated with the presence of decreased ferritin levels. Evidently, younger children are more vulnerable to lead exposure due to the increased prevalence of hand-to-mouth behavior and the time spent on the floor. This practice is especially prominent among toddlers and is supported by the findings of increased occurrence of EBLLs among 13–24 month-old children compared to infants in a recent study conducted in Greece [43]. Accordingly, we found that increasing age was inversely associated with EBLLs. The younger mean age of our immigrant compared to our refugee subpopulation is likely to be a contributory factor to the increased prevalence of EBLLs observed among them. Another factor that may partly explain the increased prevalence of EBLLs among immigrant children is that low ferritin levels were more prominent in this group. Additionally, our immigrant population mainly comprised of children from Bangladesh, a country which does not have in place a lead-screening program and where lead exposure may occur through various sources not limited to industrial discharges, but also the use of indigenous medicines, cosmetics and contaminated food and spices [44, 45]. Lastly, we noted that the probability of EBLLs was greater among children from Asia compared to those from Africa or Europe. We provided iron supplementation to deficient individuals along with oral consultation and printed leaflets in several languages covering nutritional advice and instructions of avoiding exposure to lead to the families of all children.

This is the first study to document the results of initial screening of immigrant and refugee children in a pediatric outpatient clinic, in Athens, Greece. Our findings underscore the health issues encountered by this population. This study has several limitations. Firstly, the results are restricted to a subpopulation of immigrants and refugees arriving in Athens and cannot be generalized to the whole migrant population entering Greece. Moreover, in the absence of immunization records and the difficulties in communication due to the cultural and linguistic diversity, certain information regarding this population’s past medical and vaccination history may not have been recorded. Furthermore, these results provide a snapshot of all newly arrived cases attending our clinic within a limited time period of 2010–2013 and should not be viewed as representative of the respective in subsequent years since the ethnic composition but also other factors, such as immunization coverage, may differ [46]. Lastly, our study lacks a control group comprising of children residing in Greece, to examine if any of our observations were more or less prevalent among the migrant than the native children. Nevertheless, the above limitations are outweighed by the importance of our findings and the non-limiting set of characteristics of our patient population as all newly arrived children attending the clinic within the specified period were included.

## Conclusions

In this study, we found that new immigrant and refugee children arriving in Greece commonly lack immunization records, have poor dental health, present suboptimal serologic protection against hepatitis B, but no evidence of chronic infection, elevated blood lead, eosinophilia and low ferritin levels. Many of these conditions are manageable, and if undiagnosed or left untreated, could lead to significant unfavorable health outcomes. These findings provide a basis upon which priorities could be established concerning the health screening of this vulnerable population. We recommend that all newly arriving immigrants and refugees receive a comprehensive health evaluation, including physical examination, assessment of vaccination coverage to schedule catch-up immunizations as well as screening for tuberculosis, the latter to prevent disease progression. Laboratory screening for anemia and lead exposure, especially in younger children and those originating from Asia, could prove useful. Evaluation of serologic markers against hepatitis B virus may be considered depending on the setting and resources but should not delay the administration of a multivalent vaccine dose, including hepatitis B antigen, at the time of the first visit, to optimize compliance and ensure simultaneous protection against multiple infectious diseases.

The volume, speed, and diversity of migration in Greece amid the current socioeconomic crisis are additional challenges we face in providing access to health care services to all migrants. It is evident that collaboration with non-governmental institutions and health providers at national as well as at international levels is essential. Only the joint involvement of all stakeholders could lead to improvement of the monitoring of migrant health, which in turn is imperative for the safer integration of this population and establishment of healthier communities.

## Abbreviations

anti-HBs: Antibodies against hepatitis B surface antigen
BCG: Bacillus Calmette–Guérin
BLLs: Blood lead levels
BMI: Body mass index
CDC: Centers for disease control and prevention
CI: Confidence interval
CXR: Chest X-Ray
EBLLs: Elevated blood lead levels
HBsAg: Hepatitis B surface antigen
HBV: Hepatitis B Virus
I: Immigrant
IGRAs: Interferon-gamma release assays
IOM: International Organization for Migration
OR: Odds ratios
R: Refugee
TB: Tuberculosis
